# Matricellular Protein Periostin Mediates Intestinal Inflammation through the Activation of Nuclear Factor κB Signaling

**DOI:** 10.1371/journal.pone.0149652

**Published:** 2016-02-18

**Authors:** Seong-Joon Koh, Younjeong Choi, Byeong Gwan Kim, Kook Lae Lee, Dae Woo Kim, Jung Ho Kim, Ji Won Kim, Joo Sung Kim

**Affiliations:** 1 Department of Internal Medicine, Division of Gastroenterology, Seoul National University Boramae Hospital, Seoul National University College of Medicine, Seoul, Korea; 2 Department of Otorhinolaryngology-Head and Neck Surgery, Seoul National University Boramae Hospital, Seoul National University College of Medicine, Seoul, Korea; 3 Department of Pathology, Seoul National University Boramae Hospital, Seoul National University College of Medicine, Seoul, Korea; 4 Department of Internal Medicine and Liver Research Institute, Seoul National University College of Medicine, Seoul, Korea; Massachusetts General Hospital, UNITED STATES

## Abstract

Periostin is a matricellular protein that interacts with various integrin molecules on the cell surface. Although periostin is expressed in inflamed colonic mucosa, its role in the regulation of intestinal inflammation remains unclear. We investigated the role of periostin in intestinal inflammation using *Postn*-deficient (*Postn*^-/-^) mice. Intestinal epithelial cells (IECs) were transfected by *Postn* small interfering RNAs. Periostin expression was determined in colon tissue samples from ulcerative colitis (UC) patients. Oral administration of dextran sulfate sodium (DSS) or rectal administration of trinitrobenzene sulfonic acid, induced severe colitis in wild-type mice, but not in *Postn*^-/-^ mice. Administration of recombinant periostin induced colitis in *Postn*^-/-^ mice. The periostin neutralizing-antibody ameliorated the severity of colitis in DSS-treated wild-type mice. Silencing of *Postn* inhibited inteleukin (IL)-8 mRNA expression and NF-κB DNA-binding activity in IECs. Tumor necrosis factor (TNF)-α upregulated mRNA expression of *Postn* in IECs, and recombinant periostin strongly enhanced IL-8 expression in combination with TNF-α, which was suppressed by an antibody against integrin α_v_ (CD51). Periostin and CD51 were expressed at significantly higher levels in UC patients than in controls. Periostin mediates intestinal inflammation through the activation of NF-κB signaling, which suggests that periostin is a potential therapeutic target for inflammatory bowel disease.

## Introduction

Ulcerative colitis (UC) and Crohn’s disease are two major types of inflammatory bowel disease (IBD). IBD is associated with chronic inflammation of the digestive tract, resulting in abdominal pain, persistent diarrhea, and hematochezia [1]. The prevalence of Crohn’s disease and UC in North America and some European countries is approaching 200–238 individuals per 100,000 [2]. In addition, IBD has rapidly emerged in various Asian countries including South Korea, China, and India, reducing the quality of life for affected individuals, and placing great burden of these countries [3,4]. Anti-tumor necrosis factor (TNF) agents provide beneficial effects with respect to inducing and maintaining IBD remision [5]. However, these agents have certain limitations, such as a lack of primary response and/or the loss of treatment response in some patients [6]. Major challenges remain in the development of new agents for the treatment of IBD.

Periostin, a member of the fascilin family, is a matricellular protein encoded by the *Postn* gene that interacts with various integrin molecules on cell surface [7]. Periostin participates in the development of bone, teeth, and heart vessels [8,9]. In addition, it is involved in cutaneous tissue remodeling and tumor development [10,11]. Recently, periostin was shown to promote allergic inflammation through its interaction with α_v_ integrin, as periostin activates nuclear factor kappa B (NF-κB) signaling in keratinocytes [12]. Results from a previous study showed that periostin expression is increased in the lamina propria of UC patients [13]. These results suggest that periostin is linked to intestinal inflammation and tissue repair in IBD. However, a role of periostin in the regulation of intestinal inflammation and in the pathogenesis of IBD remains unclear.

Intestinal epithelial cells (IECs) are critical for the regulation of intestinal homeostasis [14]. IECs form a mechanical barrier that protects the host from abnormal antigens, including harmful bacteria [14,15]. In addition, IECs produce chemokines such as interleukin (IL)-8 and chemokine ligand (CCL)-25, which control the migration of T cells and granulocytes [16]. IECs also produce activated tumor growth factor (TGF)-β, which can reduce T cell responses in the lamina propria [17]. Furthermore, IEC stimulation activates NF-κB, also a key signal transduction molecule in intestinal inflammation, which regulates genes associated with cytokine production, epithelial permeability, and cellular apoptosis [18].

In the present study, we sought to elucidate the mechanisms by which intestinal inflammation is mediated by periostin. We investigated the effects of genetic *Postn* deficiency in two models of murine colitis. Oral administration of DSS, or rectal administration of TNBS, induced severe colitis in wild-type mice, but not in *Postn*-deficient (*Postn*^-/-^) mice. Administration of recombinant periostin induced colitis in *Postn*^-/-^ mice. A periostin-neutralizing antibody (nAb) significantly attenuated intestinal inflammation. Knockdown of *Postn* by small interfering RNAs (siRNAs) suppressed the expression of proinflammatory cytokines in intestinal epithelial cells (IECs) through the inhibition of NF-κB signaling. These results support a role for periostin in mediating intestinal inflammation through NF-κB signaling in IECs.

## Materials and Methods

### Mice

*Postn*^-/-^ mice (male mice, 7–8 weeks) with a C57/BL6 background were kindly donated by Professor D.W. Kim of Seoul National University Boramae Hospital. Age- and gender-matched wild-type littermates (C57BL/6NCrljBgi, male mice, 7–8 weeks) were purchased from Orient (Seongnam, Korea). Mice were 7–8 weeks of age at the time of experiments. All mice were kept in specific pathogen-free housing under standard conditions of humidity and temperature with a 12-hour light/dark cycle. Euthanasia was achieved by isoflurane inhalation. Mice were euthanized according to a protocol for early endpoints when mice exhibited severe weight loss of 25% of their pre-experimental body weight. However, there were no mice died prior to the experimental endpoint.

### Induction and Evaluation of Dextran Sulfate Sodium (DSS)-induced Acute Murine Colitis

DSS (MP Biochemical, Irvine, CA; molecular weight: 35,000–50,000) was used to induce acute colitis, as described previously [19]. DSS was dissolved in drinking water to a final concentration of 4% (w/v) and administered for 5 days. The disease activity index (DAI; range from 0 to 12) was assessed daily by determining the body weight reduction, stool consistency, and rectal bleeding of each mouse. Mice were euthanized and colon tissues harvested; proximal and distal colons were fixed in 10% buffered formalin and embedded in paraffin. Sections were then stained with hematoxylin-eosin. Histological analysis was performed by two experts in a blinded manner, using a quantitative evaluation form as described previously. Briefly, we used three independent parameters, including the severity of inflammation (range from 0 to 3), the depth of inflammation (range from 0 to 3), and crypt damage (range from 0 to 4). These scores were quantified as to the percentage of tissue involvement (range from 1 to 4) [20].

### Induction and Evaluation of Trinitrobenzene sulfonic acid (TNBS)-induced colitis in mice

To induce colitis, 2.5% (w/v) TNBS solution (100 μL; Sigma-Aldrich, St. Louis, MO) in 50% ethanol was administered into the colon of *Postn*^-/-^ and wild-type mice using a thin round-tip needle attached to a 1-mL syringe. Mice were anesthetized, and the round-tip needle was carefully inserted approximately 4.0 cm from the anus, and TNBS injected. Mice were held in a vertical position for 30 s after the TNBS injection [21]. Mice were euthanized and colon tissue was harvested 4 days after the administration of TNBS. Histological damage was scored by two experts in a blinded manner using on a scale of 0–4, using previously described criteria: 0, no significant lesions; 1, minimal to mild inflammatory infiltrates in the lamina propria or the submucosa; 2, mild to moderate inflammatory infiltrates, edema occasional limited focal ulceration; 3, moderate to marked transmural inflammation, edema, necrosis in up to 70% of the mucosa; and 4, marked transmural inflammation, edema, necrosis affecting 70–100% of the mucosa [22].

### Immunohistochemical Staining of Mouse Colon Tissues

Immunohistochemistry for anti-phosphorylated-IκB kinase (pIKK)-α/β and periostin was performed on mouse colons as previously described [23]. Rabbit polyclonal anti-IKK-α/β antibody (dilution 1:100; Cell Signaling Technology, Beverly, MA) and anti-periostin antibody (dilution 1:100; Santa Cruz Biotechnology) were used as primary antibodies. Based on reaction intensity in each slide, immunoreactivity for pIKK-α/β was evaluated on a scale of 0 to 4+, as previously described [23].

### Administration of Recombinant Periostin in *Postn*^-/-^ Mice

DSS was dissolved in drinking water to a final concentration of 4% (w/v) and administered to mice for 5 days. Recombinant mouse periostin (R&D Systems, Minneapolis, MN) was dissolved in 100 μL of phosphate-buffered saline (PBS) and intraperitoneally administered every second day, commencing on the day of DSS administration (day 0). Control mice were intraperitoneally injected with 100 μL of vehicle according to the same schedule.

### Administration of Periostin nAb in Wild-Type Mice

DSS was dissolved in drinking water to a final concentration of 4% (w/v) and administered to mice for 5 days. A periostin nAb (10 μg; R&D Systems) was dissolved in 100 μL of PBS and intraperitoneally administered to mice each day for 5 days, commencing on the day of DSS administration (day 0). Control mice were administered a mouse IgG isotype control antibody (10 μg; R&D Systems) each day for 5 days.

### Cell Culture and Preparation

The human intestinal epithelial cell lines, COLO205 (American Type Culture Collection, Rockville, MD) was used between passages 15 and 30, and cultured as described previously [19]. Silencing of *Postn* was conducted by transfecting COLO205 cells with siRNAs specific for human *Postn* (Santa Cruz Biotechnology, Santa Cruz, CA) for 24 h. Scrambled siRNAs (Santa Cruz Biotechnology) were used as controls during transfection. Cells were then stimulated with TNF-α for an appropriate period. Recombinant human periostin (R&D Systems) was dissolved in PBS. COLO205 cells in the presence or absence of TNF-α were treated with two concentrations of recombinant human periostin (100 μg/mL and 500 μg/mL). An antibody against integrin α_v_ (BioLegend, San Diego, CA) was also dissolved in PBS and cells were treated with two concentrations of the integrin α_v_ antibody (10 μg/mL and 50 μg/mL).

### Real-time Reverse Transcription–Polymerase Chain Reaction (RT-PCR) and Enzyme-Linked Immunosorbent Assay (ELISA)

Real time RT-PCR was performed as described previously [23]. Total RNA was isolated from COLO205 cells using RNeasy Plus mini kit (Qiagen, Valencia, CA). One microgram of total extracted RNA was reverse transcribed using the SYBR Green PCR Master Mix and an ABI Prism 7000 Sequence Detection System (Applied Biosystems, Foster City, CA). The primers used for human IL-8, periostin, and β-actin were as follows: IL-8, (5ʹ-AAA CCA CCG GAA GGA ACC AT-3ʹ and 5ʹ-CCT TCA CAC AGA GCT GCA GAA A-3ʹ); periostin, (*Postn;* 5ʹ-ACT CTT TGC TCC CAC CAA TG-3ʹ and 5ʹ-AGA TCC GTG AAG GTG GTT TG-3ʹ); β-actin, (5ʹ-ACG GGG TCA CCC ACA CTG TGC CCA TCT A-3ʹ and 5ʹ- CTA GAA GCA TTG CGG TGG ACG ATG GAG GG-3ʹ). Each sample was assayed three times. Relative gene expression changes were calculated by normalization of the level of the target gene to the level of β-actin. The secretion of IL-8 by COLO205 cells was measured using an ELISA Kit (Invitrogen, CA) according to the manufacturer’s instructions.

### Electrophoretic Mobility Shift Assay (EMSA)

We performed EMSAs as described previously [23]. Nuclear extracts were prepared from COLO205 cells using a Nuclear Extraction Kit (Promega, Madison, WI). The concentration of proteins in samples was measured using a Bradford assay (Bio-Rad, Hercules, CA). The EMSA for NF-κB was performed using a commercially available kit (Promega).

### Western blot analysis

Western blot analysis was performed as described previously [23]. Anti-IκBα (Cell Signaling), Phospho-IκB (Cell Signaling), and β-actin (Cell Signaling) were used as primary antibodies. Target proteins were detected using the Luminescent Image Analyzer, LAS 4000 (Fuji Film, Tokyo, Japan).

### Human Colon Tissue Samples

Frozen specimens of ulcerative colitis (n = 10) and normal colorectal mucosa (n = 5) were obtained from Seoul National University Boramae Hospital. The tissue samples were de-identified and analyzed anonymously. The diagnosis of UC was based on clinical, endoscopic, histological, and radiological criteria [24,25]. Immunohistochemistry with a rabbit polyclonal antibody against periostin (dilution 1:2000; Abcam, Cambridge, MA) and anti-integrin α_v_ antibody (dilution 1:150; Abcam) was performed on formalin-fixed paraffin-embedded endoscopic biopsy tissues. Immunohistochemical expression of periostin and integrin α_v_ in colorectal mucosa tissues with or without UC was evaluated by an experienced gastrointestinal pathologist. For the assessment of periostin immunoreactivity, we evaluated the intensity of periostin expression on the border between the surface epithelium and lamina propria because previous studies demonstrated that periostin promotes an inflammatory process by interaction with integrins in epithelial cells. For each slide, reaction intensity for periostin was evaluated on a scale of 0 to 3+ (score 0, negative; score 1, weak positive; score 2, moderate positive; score 3, strong positive).

### Statistical Analysis

The data are expressed as the mean ± SD. Differences between groups were analyzed based on the Mann Whitney U test. *P* values of less than 0.05 were considered statistically significant.

### Study Approval

All mouse procedures were approved by the Institutional Animal Care and Use Committee of Seoul National University Boramae Hospital. Human studies were approved by the ethics committee of Seoul National University Boramae Hospital (Protocol Number: 26-2014-81). Our institutional review board waived the need for consent.

## Results

### Periostin Mediates Intestinal Inflammation in Murine Models of Colitis

**Fig 1:**
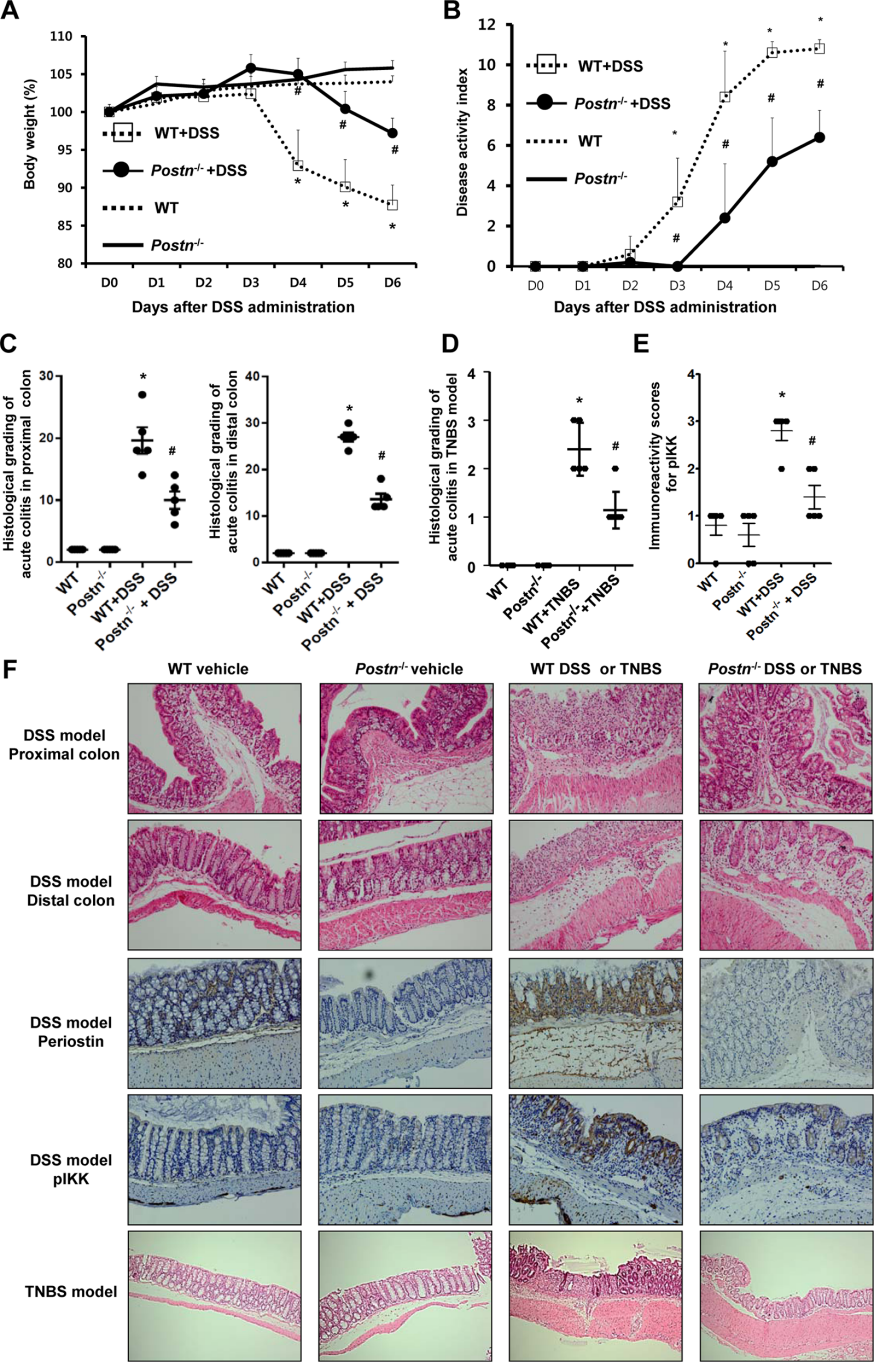
Periostin mediates intestinal inflammation in murine models of colitis. (A and B) Mice were divided into four groups in the DSS-induced colitis model (n = 5): wild-type control, *Postn*^-/-^ control, wild-type treated with DSS, or *Postn*^-/-^ treated with DSS. *Postn*^-/-^ mice showed significant attenuation in the body weight reduction and the disease activity index three days after DSS administration. (C) Histological evaluations of the colons in the DSS-induced acute murine colitis model. The total histological scores were derived from the severity and extent of inflammation and crypt damage (mean ± SD). Results are representative of at least three separately examined sites. (D) Histological evaluations of colons in the TNBS-induced colitis (mean ± SD). Mice were divided into four groups in the TNBS-induced colitis model: wild-type control (n = 4), *Postn*^-/-^ control (n = 4), wild-type treated with TNBS (n = 5), or *Postn*^-/-^ treated with TNBS (n = 7). Results are representative of at least three separately examined sites. (E) Immunoreactivity index for phosphorylated IκB kinase (pIKK)-α/β (mean ± SD) of the colonic epithelium in DSS-induced murine colitis. DSS exposure in wild-type mice induced pIKK activity in the colonic epithelium. Periostin deficiency significantly attenuated phosphorylated pIKK-α/β activity in the colonic mucosa. (F) Representative H&E staining and immunostained images of pIKK-α/β and periostin in the colon tissues (Magnification x 200). These results are representative of three independent experiments. WT, wild-type; *Postn*^-/-^, *Postn*-deficient; DSS, dextran sulfate sodium; TNBS, trinitrobenzene sulfonic acid; pIKK, phosphorylated-IκB kinase *P < 0.05 compared with wild-type mice without DSS or TNBS, ^#^ *P* < 0.05 compared with wild-type mice treated with DSS or TNBS.

To investigate whether periostin mediates intestinal inflammation, we conducted an *in vivo* study using a DSS-induced acute colitis model. Wild-type mice showed a substantial loss in body weight after 3 days of DSS exposure. However, this reduction in body weight was attenuated in *Postn*^-/-^ mice (Fig 1A). In addition, the DAI for *Postn*^-/-^ mice was attenuated 3 days after DSS administration (Fig 1B). Mice were euthanized 6 days after DSS administration, and the gross appearance of the colon examined. A marked reduction in colon length was seen in wild-type mice exposed to DSS as compared with wild-type controls, indicating that severe colitis was induced in the former. However, shortening of the colon was significantly attenuated in *Postn*^-/-^ mice treated with DSS (data not shown).

Histopathological analysis revealed severe inflammation in the proximal and distal colon of wild-type mice exposed to DSS. In these mice, entire intestinal crypts were destroyed, and increased inflammatory cell infiltration was observed. In addition, crypt abscesses and extensive edema of the submucosa were found. However, for *Postn*^-/-^ mice exposed to DSS, we observed reduced histological grades in the proximal and distal colon. No clinical or histological change was shown in *Postn*^-/-^ mice that were not exposed to DSS, similar to what was observed for wild-type mice that were not exposed to DSS (Fig 1C and 1F).

NF-κB signaling in IECs plays a critical role in the regulation of intestinal inflammation. Therefore, we performed immunohistochemistry using an antibody against pIKK-α/β. In wild-type mice exposed to DSS, pIKK activity was induced in the colonic epithelium. However, a deficiency in periostin significantly attenuated pIKK activity in the colonic mucosa (Fig 1E and 1F). We measured periostin expression in the lamina propria of wild-type mice with DSS exposure. The normal colon of wild-type mice showed periostin expression in the lamina propria and DSS exposure significantly increased periostin expression (Fig 1F).

To confirm the effects of periostin deficiency in type 1 helper T cell (Th1)-mediated murine colitis, we conducted experiments using a TNBS-induced colitis model. Intrarectal administration of TNBS induced severe inflammation, including edema, ulceration, and transmural inflammation. However, *Postn*^-/-^ mice exposed to TNBS exhibited reduced severity of intestinal inflammation, which was statistically significant (Fig 1D and 1F).

### Administration of Recombinant Periostin Induces Colitis in *Postn*^-/-^ Mice

**Fig 2:**
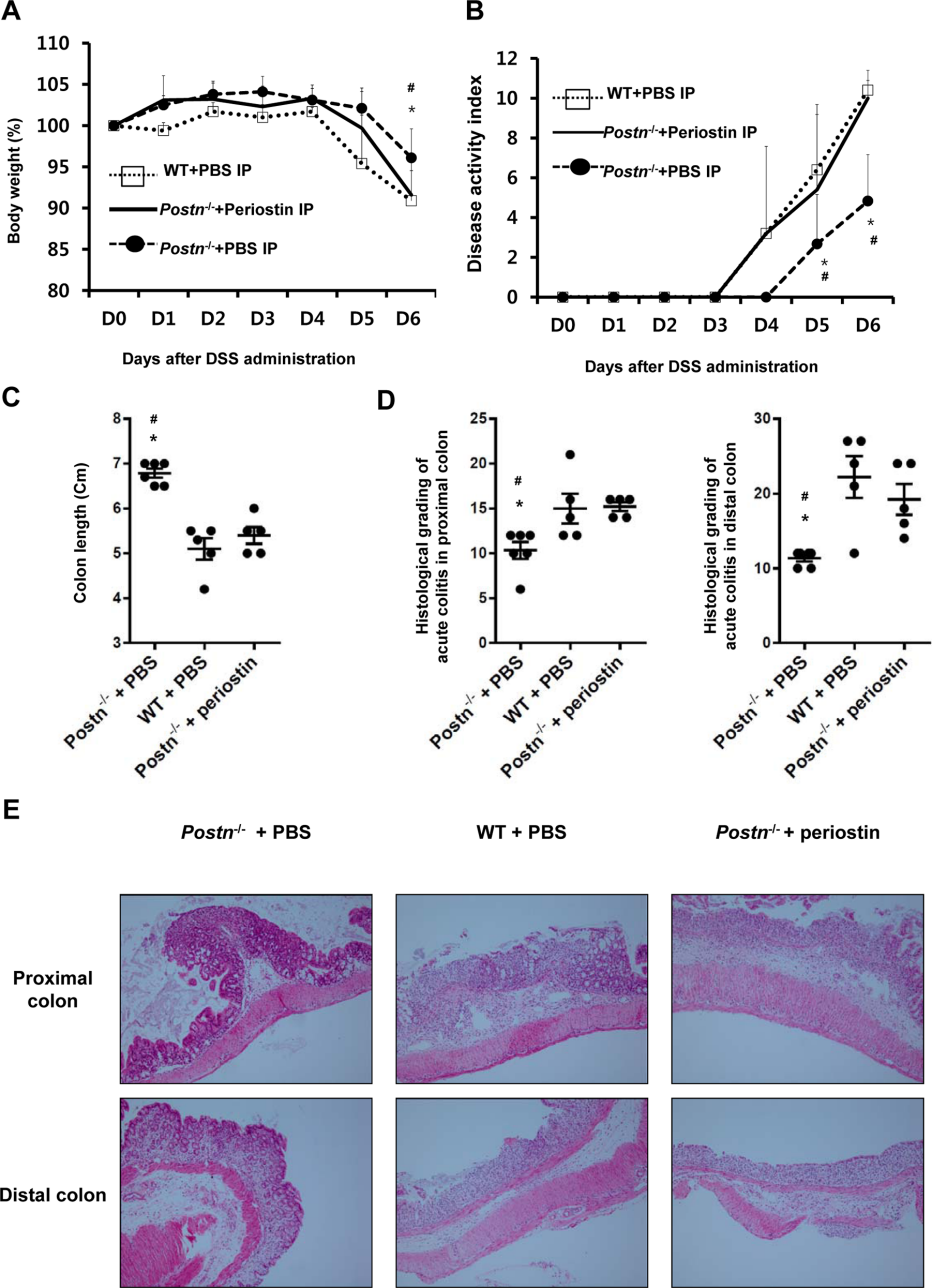
Administration of recombinant periostin induces colitis in *Postn*^-/-^ Mice. Mice were divided into three groups (n = 5): wild-type treated with PBS, *Postn*^-/-^ treated with PBS, or *Postn*^-/-^ treated with recombinant periostin (10 μg). Recombinant mouse periostin was dissolved in 100 μL of phosphate-buffered saline (PBS) and intraperitoneally administered every second day, commencing on the day of DSS administration (day 0). Control mice were intraperitoneally injected with 100 μL of vehicle according to the same schedule. (A) Body weight change after DSS administration. (B) Changes in the disease activity index after DSS administration. Administration of recombinant periostin resulted in a significant reduction in body weight, along with increased DAI when compared with that seen for control *Postn*^-/-^ mice treated with PBS. (C) Extracted colon length at day 6 after DSS administration. (D) Histological evaluations of colons in the DSS-induced acute murine colitis model (mean ± SD). The total histological scores were derived from the severity and extent of inflammation and crypt damage. (E) Results are representative H&E staining of at least three separately examined sites (Magnification x 100). These results are representative of two independent experiments. WT, wild-type; *Postn*^-/-^, *Postn*-deficient; PBS, phosphate-buffered saline; IP, intraperitoneal injection; DSS, dextran sulfate sodium *P < 0.05 compared with *Postn*^-/-^ mice treated with recombinant periostin, ^#^ *P* < 0.05 compared with wild-type mice treated with DSS.

To confirm a role of periostin in mediating colitis, we investigated whether external supplement of recombinant mouse periostin exacerbates the severity of colitis in *Postn*^-/-^ mice. Administration of recombinant periostin resulted in a significant reduction in body weight, along with increased DAI when compared with that seen for control *Postn*^-/-^ mice treated with PBS (Fig 2A and 2B). In addition, severe colon shortening was seen in *Postn*^-/-^ mice given recombinant periostin (Fig 2C). There were no significant differences in body weight reduction, DAI, and colon length between wild-type mice and *Postn*^-/-^ mice that were administered recombinant periostin. Recombinant periostin induced severe colitis in *Postn*^-/-^ mice, resulting in histological damage similar to that seen in the proximal and distal colon of wild-type mice (Fig 2D and 2E).

### Periostin nAb Attenuates Acute Murine Colitis in Wild-Type Mice

**Fig 3:**
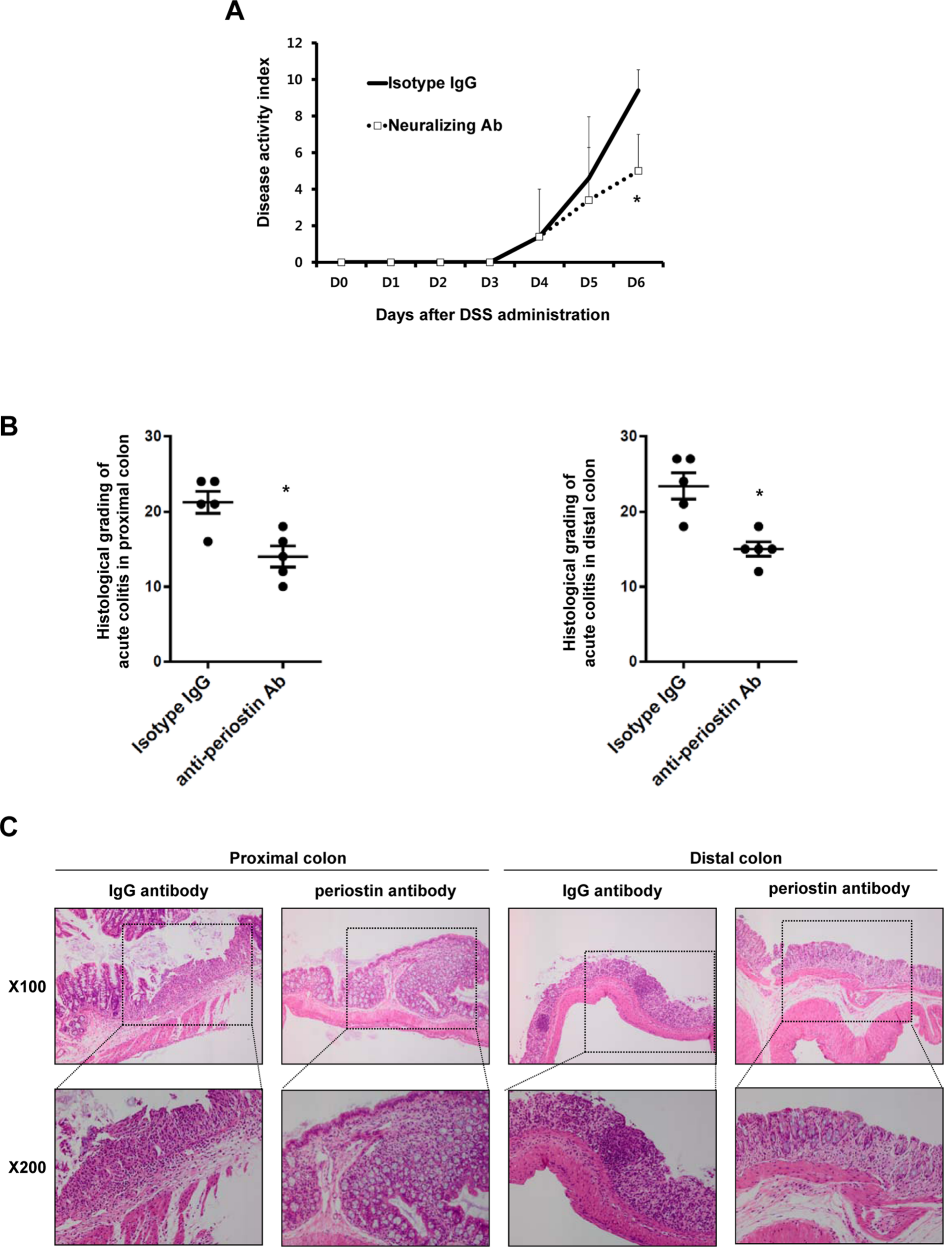
Periostin-neutralizing antibody attenuates acute murine colitis in wild-type mice. Mice were divided into two groups (n = 5). A periostin-neutralizing antibody (nAb) was dissolved in 100 μL of PBS and intraperitoneally administered to mice each day for 5 days, commencing on the day of DSS administration (day 0). Control mice were administered a mouse IgG isotype control antibody each day for 5 days. (A) Change in the disease activity index after DSS administration. Periostin nAb significantly attenuated disease activity index, compared with mice treated with isotype IgG control antibody. (B and C) The total histological scores were derived from the severity and extent of inflammation and crypt damage (mean ± SD). Results are representative of at least three separately examined sites. These results are representative of two independent experiments. WT, wild-type; *Postn*^-/-^, *Postn*-deficient; PBS, phosphate-buffered saline; Isotype IgG, Isotype IgG control antibody; anti-periostin Ab, periostin-neutralizing antibody; DSS, dextran sulfate sodium; X100, magnification x100; X200, magnification x200 * *P* < 0.05 compared with isotype IgG control antibody.

To confirm a role of periostin in intestinal inflammation and to address potential issues regarding *Postn*^-/-^ mice, we investigated the effects of a nAb against periostin in acute murine colitis. The periostin nAb significantly attenuated the severity of colitis, as shown by the DAI (Fig 3A). Gross colon appearance revealed severe shortening in mice administered an IgG isotype antibody. However, colon shortening was significantly attenuated in mice treated with the periostin nAb. Histologic grading also showed that the periostin nAb resulted in reduced overall colonic damage as compared with that seen in mice treated with the isotype IgG (Fig 3B and 3C). Collectively, these results suggest that blocking the activity of periostin with a nAb can attenuate intestinal inflammation in a murine model of colitis.

### *Postn* Silencing Reduces IL-8 Expression by Inhibiting NF-κB Signaling in IECs

**Fig 4:**
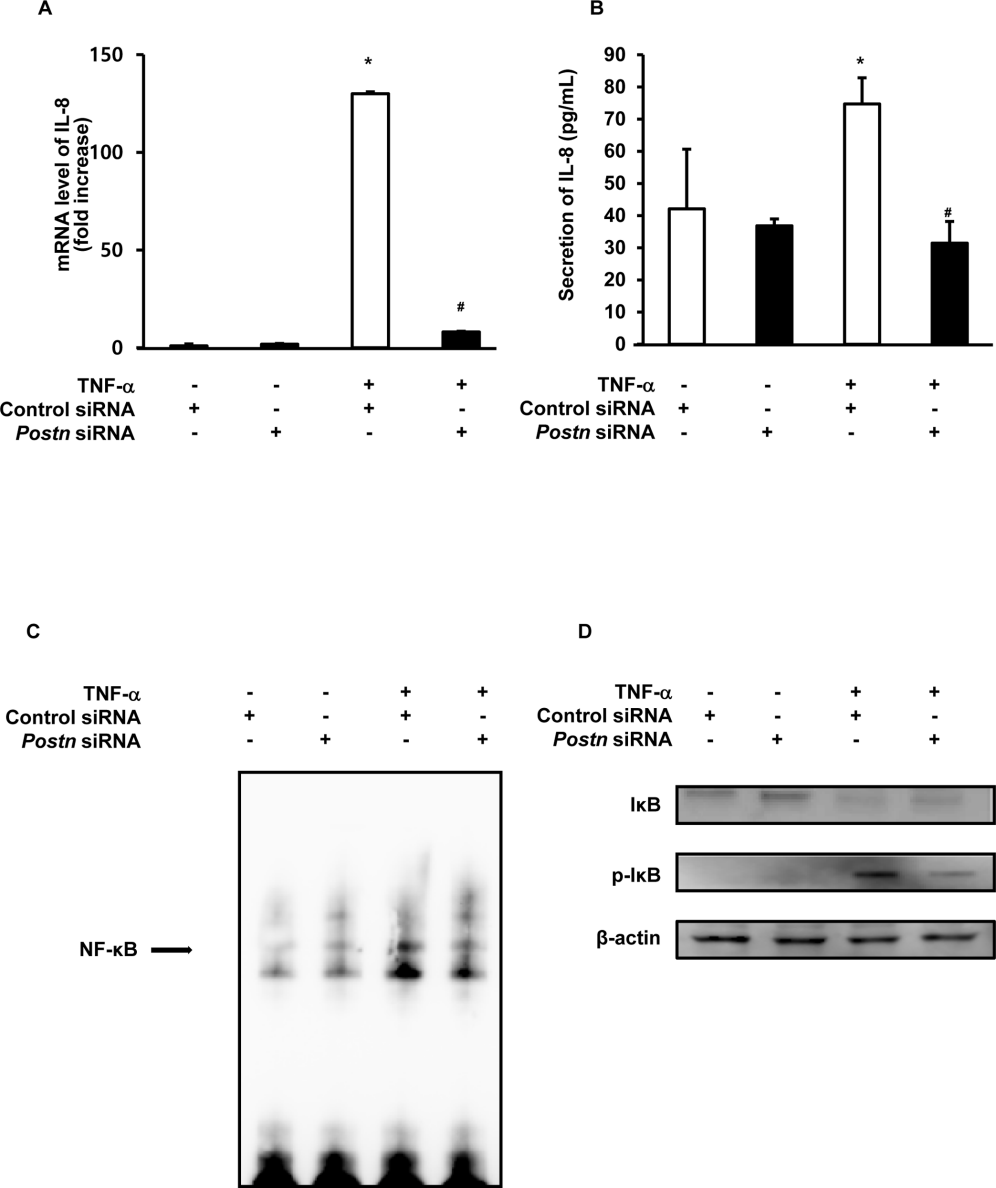
*Postn* silencing reduces IL-8 expression by inhibiting NF-κB signaling in intestinal epithelial cells. COLO205 cells were transfected with control or *Postn*-specific siRNA for 24 h. (A) COLO205 cells were stimulated with TNF-α (10 ng/mL) for 4 h. IL-8 mRNA expression was measured by real-time RT-PCR. Levels were normalized to β-actin. Data are expressed as fold-change in mRNA transcript levels relative to the unstimulated control (mean ± SD, n = 3). (B) COLO205 cells were stimulated with TNF-α (10 ng/ml) for 24 h. Secretion of IL-8 was measured by ELISA (mean ± SD, n = 3). (C) COLO205 cells were stimulated with TNF-α (10 ng/ml) for 30 min. NF-κB DNA binding activity in the nuclear extracts was assessed by electrophoretic mobility shift assay. TNF-α, tumor necrosis factor-α; siRNA, small interfering ribonucleic acid * *P* < 0.05 compared with control siRNA without TNF-α stimulation, ^#^ *P* < 0.05 < compared with control siRNA stimulated with TNF-α.

Because our *in vivo* data showed that a deficiency in periostin inhibited pIKK activity in the colonic epithelium, we evaluated the effects of *Postn* silencing on NF-κB signaling in IECs. Stimulation of COLO205 cells with TNF-α for 4 h resulted in an approximately 130-fold increase in the expression level of the gene encoding IL-8, a downstream target and surrogate marker for NF-κB signaling, compared with that seen for unstimulated cells. Transfection of COLO205 cells with *Postn*-specific siRNAs strongly suppressed IL-8 mRNA expression and protein secretion in COLO205 cells (Fig 4A and 4B). We then conducted EMSAs to evaluate the consequence of *Postn* inhibition on NF-κB DNA-binding activity. Stimulation with TNF-α resulted in an increase in NF-κB DNA-binding activity. However, the application of *Postn* siRNAs significantly reduced NF-κB DNA-binding activity in COLO205 cells (Fig 4C). Finally, *Postn*-specific siRNAs strongly suppressed TNF-α-induced IκBα phosphorylation and recovered IκBα degradation (Fig 4D).

### Periostin Enhances Proinflammatory Cytokine Production via Interaction with Integrin α_v_ in IECs

**Fig 5:**
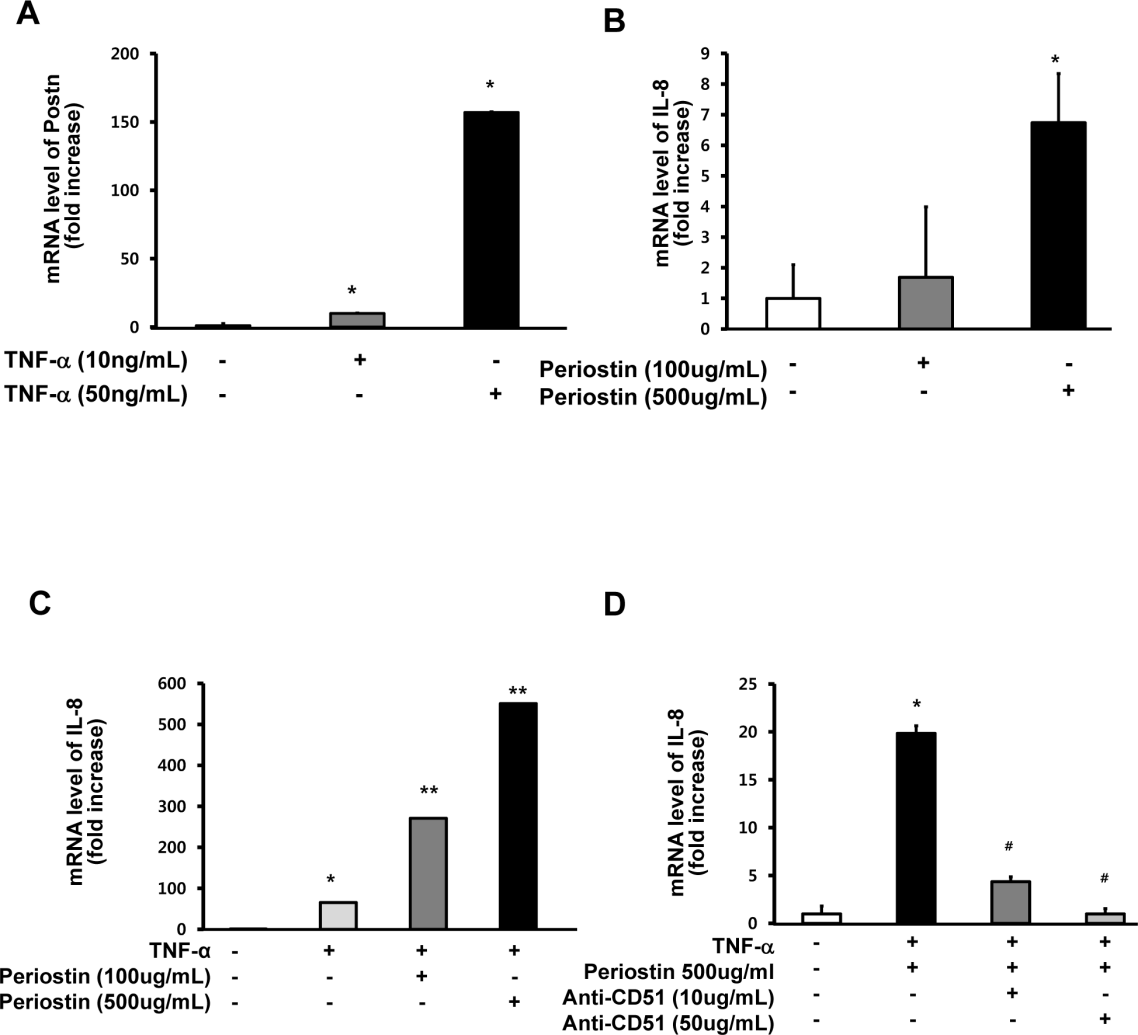
Periostin enhances proinflammatory cytokine production via interaction with integrin in intestinal epithelial cells. (A) COLO205 cells were stimulated with TNF-α (10 ng/mL or 50 ng/mL) for 4 h. *Postn* mRNA expression was measured by real-time RT-PCR. Levels were normalized to β-actin. Data are expressed as fold-change in mRNA transcript levels relative to the unstimulated control (mean ± SD, n = 3). (B) COLO205 cells were stimulated with recombinant periostin (100 μg/mL or 500 μg/mL) for 24 h. IL-8 mRNA expression was measured by real-time RT-PCR (mean ± SD, n = 3). (C) COLO205 cells were pretreated with recombinant periostin for 24 h and then stimulated with TNF-α (10 ng/mL) for 4 hr. IL-8 mRNA expression was measured by real-time RT-PCR (mean ± SD, n = 3). (D) COLO205 cells were pretreated with anti-integrin α_v_ (CD51) antibody and recombinant periostin for 24 h and then stimulated with TNF-α (10 ng/mL) for 4 h (mean ± SD, n = 3). TNF-α, tumor necrosis factor-α; Anti-CD51, anti-integrin α_v_ antibody * *P* < 0.05 compared with negative control (without stimulation), ** *P* < 0.05 compared with TNF-α stimulation alone, ^#^ *P* < 0.05 compared with co-stimulation with TNF-α and recombinant periostin.

We sought to determine whether periostin increases proinflammatory cytokine production through interactions with IECs. Stimulation with TNF-α significantly increased expression of *Postn* in IECs (Fig 5A). Recombinant periostin upregulated IL-8 expression in IECs (Fig 5B), and treatment of IECs with recombinant periostin in combination with TNF-α resulted in a synergistic increase in IL-8 expression (Fig 5C). However, the enhanced production of IL-8 expression by co-stimulation with TNF-α and recombinant periostin was significantly inhibited by an antibody against integrin α_v_, in a dose dependent manner (Fig 5D).

### Periostin is Highly Expressed in the Colon Tissues of UC Patients

**Fig 6:**
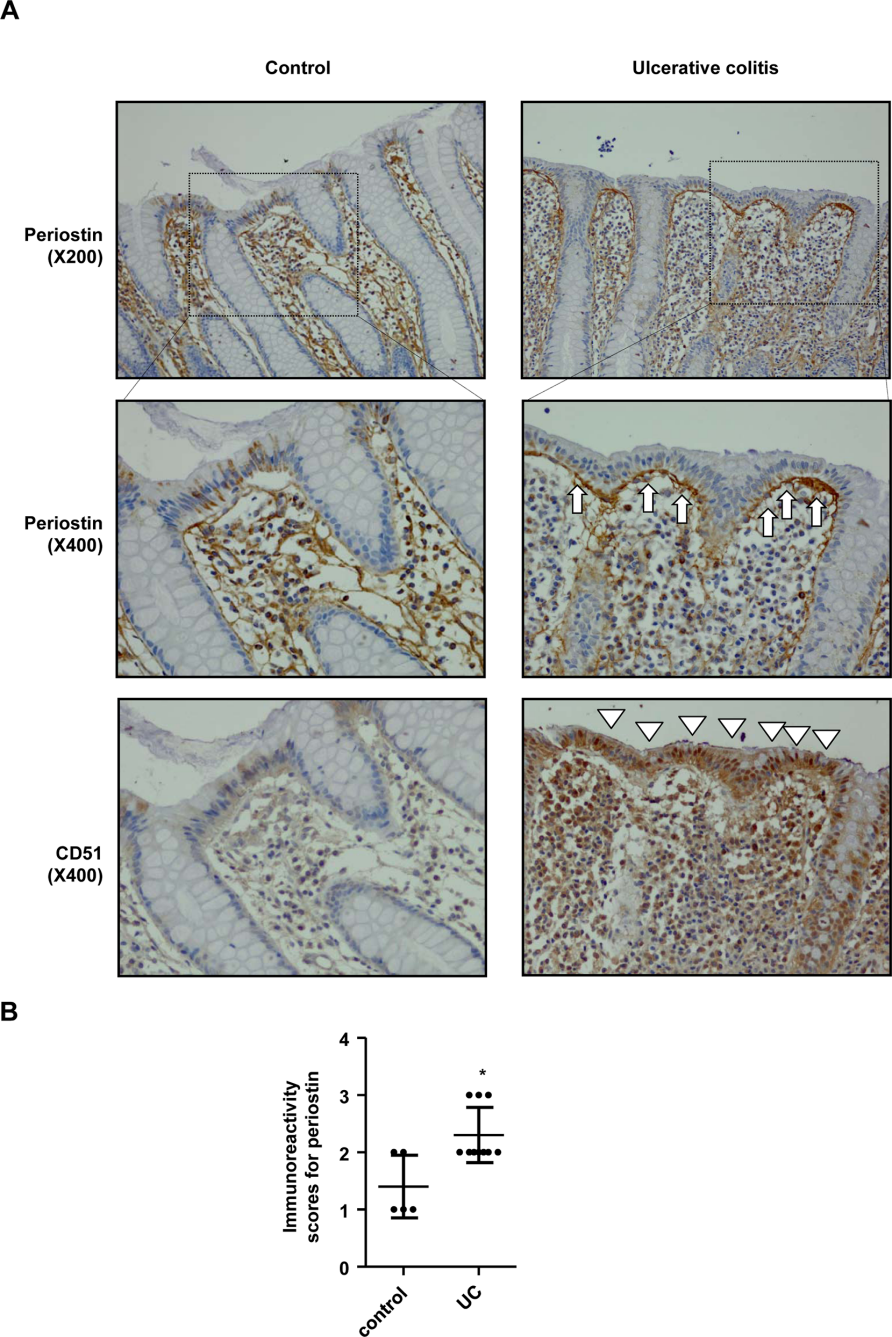
Periostin is highly expressed in the colon tissues of ulcerative colitis patients. **Tissue specimens were stained immunohistochemically with periostin and integrin α**_**v**_ **(CD51).** (A) Representative immunostained images of periostin in normal subjects and UC patients. Patients with ulcerative colitis exhibited linear staining of periostin along the subepithelial border (arrow), and a simultaneous increase in the immunoreactivity of CD51, periostin’s functional receptor, in the colonic epithelium where periostin was expressed (arrowhead). (B) The scores of periostin immunoreactivity were significantly increased in colonic samples of ulcerative colitis patients (mean ± SD). CD51, integrin α_v_; UC, ulcerative colitis; X200, magnification x 200; X400, magnification x 400 * *P*<0.05 compared with controls.

Periostin expression was observed in the lamina propria of both UC patients and controls. Interestingly, the intensity of periostin immunoreactivity was significantly stronger, mainly at the border between the epithelium and lamina propria, in colonic samples of UC patients compared with normal controls (Fig 6A and 6B). Because the *in vitro* study showed that anti-integrin α_v_ antibody inhibited enhanced IL-8 expression by costimuation with TNF-α and recombinant periostin in IECs, we performed immunohistochemistry using anti-integrin α_v_ antibody to elucidate whether enhanced periostin expression at the subepithelial border among patients with UC directly correlates with integrin α_v_ expression in the colonic epithelium. Integrin α_v_ expression was intensified in epithelial cells along with periostin expression in patients with UC.

## Discussion

The therapeutic goals of IBD are to induce mucosal healing, and to maintain long-term clinical remission without surgery [26]. Recent advances in our understaning of the molecular biology and pathogenesis of IBD has led to the identification of various appropriate therapeutic targets, such as TNF-α [27]. It has been reported that TNF-α antagonists are effective at inducing mucosal healing and clinical remission [5,28]. However, clinical remission and mucosal healing is observed in less than one-third of IBD patients [29]. In addition, a substantial number of those patients fail to respond to these TNF-α antagonists over time [6,30]. Therefore, concerted efforts have been made to identify new therapeutic targets for IBD [31]. In the current study, we investigated the role of periostin as a new therapeutic target for IBD. We used two different murine models of IBD and found that periostin promoted intestinal inflammation. Through *in vitro* experiemnts, we established a role of periostin in mediating intestinal inflammation. The role of periostin in the pathogenesis of IBD was confirmed using tissues from UC patients, suggesting that periostin could be a potential new therapeutic target for IBD.

Periostin is critical for enhancing airway inflammation, hepatic inflammation, and fibrosis in mice [32,33]. In addition, periostin plays a role in regulating chronic allergic skin inflammation [12]. Periostin is expressed at higher levels in patients with UC than in healthy controls [13]. To elucidate the role of periostin in IBD, we used a DSS-induced colitis model to examine intestinal inflammation in *Postn*^-/-^ and wild-type mice. Clinical and histopathological evaluation of *Postn*^-/-^ mice revealed attenuated severity of colitis. To confirm our results in a Th1-mediated colitis model, we conducted experiments using a TNBS-induced colitis model in mice. We clearly showed that the oral administration of DSS, or rectal administration of TNBS, induced severe colitis in wild-type mice but not in *Postn*^-/-^ mice. In addition, the administration of recombinant periostin to *Postn*^-/-^ mice resulted in severe colitis, comparable with that seen in wild-type mice. Taken together, our findings indicate that periostin is critical for amplifying intestinal inflammation. To the best of our knowledge, this is the first study to highlight the role of periostin in the regulation of intestinal inflammation.

IECs are one of the key factors for regulating homeostasis in intestinal inflammation.[34] Additionally, complex molecular cascades are involved in the pathogenesis of intestinal inflammation [35]. NF-κB is one of the critical transcription factors involved in chronic inflammatory diseases, including IBD [36]. Results from a previous study demonstrated that periostin directly induces IL-25, a key cytokine in the initiation of the inflammatory cascade in atopic dermatitis *via* NF-κB activation in keratinocytes [12]. Therefore, we investigated a role of periostin in IECs using *in vivo* and *in vitro* models. DSS strongly induced pIKK activity in the colonic epithelium of wild-type mice. However, *Postn*^-/-^ mice showed attenuated pIKK activity in the colonic mucosa. Transfection with *Postn*-specific siRNAs resulted in decreased IL-8 expression levels and reduced NF-κB DNA-binding activity in IECs. These results indicate that a deficiency in *Postn* reduces intestinal inflammation by blocking the NF-κB pathway in IECs.

A periostin blocking agent resulted in reduced liver steatosis, by blocking an autocrine periostin loop [37]. Periostin acts as a matricellular protein by interacting with integrin molecules such as α_v_β_3_, α_v_β_5_, and α_4_β_6,_ in various cells including keratinocytes, cardiomyocytes, and endothelial cells [38,39]. However, the precise mechanism by which periostin promotes intestinal inflammation in IECs remains unknown. Our *in vivo* data showed that periostin exists in the normal colonic mucosa, and that its expression is significantly increased in the lamina propria of mice with colitis. We hypothesized that periostin in the lamina propria promotes intestinal inflammation by interacting with IECs through integrin molecules. Stimulation with TNF-α significantly increased the expression of *Postn* in IECs. Recombinant periostin directly activated the expression of IL-8 in IECs. The expression of IL-8 was synergistically increased when co-stimulated with TNF-α and periostin, which was significantly attenuated by an antibody against integrin α_v_. To highlight the functional role of periostin in IBD pathogenesis, we performed immunohistochemistry on tissue samples from UC patients. We observed linear staining of periostin along the subepithelial border, and a simultaneous increase in the immunoreactivity of integrin α_v_ in the colonic epithelium where periostin was expressed. Based on these results, we postulated that increased expression of periostin, during initial inflammatory stages, contributes to the amplification of intestinal inflammation *via* interactions with integrin molecules.

Although we demonstrated that *Postn*^-/-^ mice exhibited clinical and histopathological improvement in a DSS-induced colitis model, it remains unclear whether periostin is an effective therapeutic target. To address this issue, we showed that recombinant periostin induced severe colitis in DSS-treated *Postn*^-/-^ mice, and periostin nAb reduced colitis in DSS-treated wild-type mice, suggesting that periostin could be an effective therapeutic target for IBD. Furthermore, our *in vitro* data indicated that periostin promotes proinflammatory cytokine production in IECs when they are co-stimulated with TNF-α. Taken together, our data suggest that neutralization of periostin activity with a nAb might have a synergistic role in reducing intestinal inflammation, when used in conjunction with a TNF-α antagonist.

We showed that an anti-integrin α_v_ antibody inhibited periostin- and TNF-α-induced IL-8 mRNA expression in IECs. Integrin α_v_, periostin’s functional receptor, is highly expressed in UC patients. Blocking interactions between periostin and integrin α_v_, using an anti-integrin α_v_ antibody, attenuated skin allergic inflammation in a murine model [12]. These results suggest that periostin and CD51 might be potential therapeutic targets for IBD. However, it has been reported that the majority of integrin α_v_-deficient mice die before birth [40]. Interestingly, the loss of myeloid α_v_ integrins induces spontaneous colitis, wasting, and autoimmunity in mice [41]. Furthermore, the loss of integrin α_v_β8 on dendritic cells induces colitis in mice [42]. Therefore, use of the antibody against integrin α_v_ appears to result in severe adverse events, in comparison with the effects of the periostin nAb. Further studies are required to elucidate the efficacy and safety of blocking periostin and integrin α_v_ interactions.

In conclusion, our results provide new information regarding the effects of periostin modulation in *in vivo* and *in vitro* models of colitis. We have also provided a molecular basis of periostin action during the regulation of intestinal inflammation. Our results suggest that periostin is a potential therapeutic target for treating IBD patients.
